# Identification of Subtypes and a Delayed Graft Function Predictive Signature Based on Ferroptosis in Renal Ischemia-Reperfusion Injury

**DOI:** 10.3389/fcell.2022.800650

**Published:** 2022-02-08

**Authors:** Xiangling Wei, Weiming Deng, Zhanwen Dong, Zhenwei Xie, Jinhua Zhang, Ruojiao Wang, Rui Zhang, Ning Na, Yu Zhou

**Affiliations:** ^1^ Department of Kidney Transplantation, The Third Affiliated Hospital of Sun Yat-sen University, Guangzhou, China; ^2^ Department of Pancreatic Surgery, Department of General Surgery, Guangdong Provincial People’s Hospital, Guangdong Academy of Medical Sciences, Guangzhou, China

**Keywords:** renal ischemia-reperfusion injury, ferroptosis, signature, subtypes, consensus clustering, delayed graft function

## Abstract

Renal ischemia-reperfusion injury (IRI) is an inevitable process in kidney transplantation, leading to acute kidney injury, delayed graft function (DGF), and even graft loss. Ferroptosis is an iron-dependent regulated cell death in various diseases including IRI. We aimed to identify subtypes of renal IRI and construct a robust DGF predictive signature based on ferroptosis-related genes (FRGs). A consensus clustering analysis was applied to identify ferroptosis-associated subtypes of 203 renal IRI samples in the GSE43974 dataset. The FRG-associated DGF predictive signature was constructed using the Least Absolute Shrinkage and Selection Operator (LASSO), and its robustness was further verified in the validation set GSE37838. The present study revealed two ferroptosis-related patient clusters (pBECN1 and pNF2 cluster) in renal IRI samples based on distinct expression patterns of BECN1 and NF2 gene clusters. Cluster pBECN1 was metabolically active and closely correlated with less DGF, while pNF2 was regarded as the metabolic exhausted subtype with higher incidence of DGF. Additionally, a six-gene (ATF3, SLC2A3, CXCL2, DDIT3, and ZFP36) ferroptosis-associated signature was constructed to predict occurrence of DGF in renal IRI patients and exhibited robust efficacy in both the training and validation sets. High-risk patients tended to have more infiltration of dendritic cells, macrophages, and T cells, and they had significantly enriched chemokine-related pathway, WNT/β-catenin signaling pathway, and allograft rejection. Patients with low risks of DGF were associated with ferroptosis-related pathways such as glutathione and fatty acid metabolism pathways. In conclusion, patient stratification with distinct metabolic activities based on ferroptosis may help distinguish patients who may respond to metabolic therapeutics. Moreover, the DGF predictive signature based on FRGs may guide advanced strategies toward prevention of DGF in the early stage.

## Introduction

Kidney transplantation is the major treatment for patients with end-stage renal disease. But wide discrepancies exist between the demand and supply of kidney for transplantation, which renders us to improve success rates and survival of kidney allografts. Ischemia-reperfusion injury (IRI) is an inevitable process in kidney transplantation, during which the cold ischemia, warm ischemia, and subsequent reperfusion result in dysfunction of renal allografts (Smith et al., 2019). The major damage caused by IRI is the loss of function in tubular epithelial cells, which then contributes to acute kidney injury (AKI), delayed graft function (DGF), and loss of allografts (Saat et al., 2016). DGF increases the possibility of prolonged hospitalization or more renal failures (Schroppel and Legendre, 2014). Much interest has been devoted to the prediction of DGF, but there is still lack of an effective predictive tool. Irish et al. (2010) proposed a nomogram integrating donor’s and recipient’s risk factors to predict DGF. But it is far from satisfactory, and thus better predictive tools are needed.

Ferroptosis is an iron-dependent and regulated cell death, characterized by membrane damage due to accumulation of lipid-based reactive oxygen species (ROS) (Yang and Stockwell, 2016). The glutamate/cystine antiporter system Xc^−^, which transports cystine inside a cell to produce glutathione, would be inhibited. Subsequent over-consumption of glutathione leads to inactivation of lipid repair enzyme glutathione peroxidase 4 (GPX4) (Stockwell et al., 2017). Emerging research discovered that ferroptosis exerted profound effects on a variety of pathological processes and diseases, including cancer (Friedmann Angeli et al., 2019; Mou et al., 2019), degenerative diseases (Chen et al., 2015; Do Van et al., 2016; Guiney et al., 2017), and stroke (Alim et al., 2019). In the field of renal IRI, it was reported that inducible inhibition of GPX4 led to acute kidney failure attributed to ferroptosis (Friedmann Angeli et al., 2014). Additionally, miR-182-5p, miR-378a-3p, and PANX1 promoted ferroptosis in renal IRI (Su et al., 2019; Ding et al., 2020). In addition, pachymic acid and XJB-5-131 showed protective roles in renal IRI by inhibiting ferroptosis (Zhao et al., 2020; Jiang et al., 2021). However, current research has not analyzed ferroptosis-related gene (FRG) profiles comprehensively in renal IRI.

This study sought to identify clusters with distinct functions in IRI patients and construct a DGF predictive signature by comprehensive analysis of FRGs. We first screened differentially expressed FRGs (DFRG) in IRI patients using data from the FerrDb and GEO databases. A consensus clustering analysis distributed IRI patients into two clusters with distinct molecular traits based on FRGs. Furthermore, a robust DGF predictive signature was constructed using DFRGs and validated in the external validation set. It was noteworthy that this was the first study to establish patient classification and construct a DGF predictive signature based on FRG expression profiles in renal IRI.

## Materials and Methods

### Data Acquisition

The gene expression profiles of kidney tissues obtained after ischemia-reperfusion (IRI group) in kidney transplantation and kidney tissues obtained before retrieval (control group) were analyzed using the GSE43974 dataset from the Gene Expression Omnibus (GEO) database (http://www.ncbi.nlm.nih.gov/geo). There were 203 IRI samples and 188 control samples with clinical information in the GSE43974. Additionally, the GSE37838 comprised of 70 samples with corresponding clinical information, was used as the validation set. The probe matrices of GSE43974 and GSE37838 were matched to the platform GPL10558 and GPL570 to obtain gene symbols. Data were normalized using the RMA algorithm.

A total of 259 FRGs were retrieved from the FerrDb database (http://zhounan.org/ferrdb) (Zhou and Bao, 2020). There were 237 FRGs in the gene matrix of GSE43974, and these FRGs were used for further research.

### Differential Expression Analysis

The differential expression analysis between IRI and control in GSE43974 was performed using the “limma” package in R software (Ritchie et al., 2015). Genes with | log(fold change) | ≥ 1 and FDR adjusted *p* < 0.05 (Benjamini and Hochberg method) were considered differentially expressed genes (DEG). DFRGs were defined as common genes in both DEGs and FRGs. The volcano plot showed DFRGs between IRI and control using the “ggpubr” package in R software.

### Functional Enrichment Analysis

To clarify the function of DFRGs, the “clusterprofiler” R package was used to perform Gene Ontology (GO) and Kyoto Encyclopedia of Genes and Genomes (KEGG) functional enrichment analyses (Yu et al., 2012). Additionally, the function of the top 10 genes in each cluster was analyzed using the “clueGO” plugin of Cytoscape software 3.8.2 (Shannon et al., 2003; Bindea et al., 2009). The gene set variation analysis (GSVA), a functional enrichment analysis method for assessing pathway activity variation, was performed to determine KEGG pathway activities and metabolic pathway activities using pre-determined genesets including the KEGG geneset and the metabolic geneset. The KEGG geneset was retrieved from the GSEA website (http://www.gsea-msigdb.org/gsea/index.jsp), and the metabolic geneset was obtained from Rosario et al. (2018). Terms with adjusted *p* < 0.05 (Benjamini and Hochberg method) were selected. Gene set enrichment analyses (GSEA) in terms of signaling pathways, immune, and metabolism, were conducted in IRI samples of GSE43974, divided by high-risk and low-risk groups. Terms with a nominal *p*-value < 0.05 and an FDR q-value < 0.25 were statistically significant.

### Construction of Transcription Factor Network and LncRNA–miRNA–mRNA Network

TRRUST (version 2) is a manually curated database consisting of human and mouse transcriptional regulatory networks, derived from published articles with experimental studies of transcriptional regulation (Han et al., 2018). Transcription factors which regulated DFRGs were predicted using the TRRUST database. Then we constructed the transcription factor regulatory network that consisted of DFRGs and their targets or regulators using Cytoscape software (version 3.8.2) (Shannon et al., 2003). Additionally, miRtarBase, an experimentally validated miRNA–target interaction database, was used to predict miRNA of DFRGs (Huang et al., 2020). LncACTdb was a comprehensive database of experimentally supported competing endogenous RNA (ceRNA) interactions (Wang et al., 2019). To analyze the ceRNA network of DFRGs, miRtarBase and LncACTdb databases were used to predict miRNA and lncRNA, respectively.

### Correlation and Co-Expression Analyses

The Spearman correlation was computed among all FRGs in IRI samples of GSE43974, and genes with *p* < 0.05 were considered statistically significant. Moreover, a list of 30 genes involved in inflammation and inflammasome and 1,881 genes involved in immunity, including innate, adaptive, and cytokine signaling, were retrieved from Benfeitas et al. (2019). Then, co-expression analyses between the top 10 genes in both clusters and aforementioned genes were performed, and co-expressed genes with *p* < 0.05 were selected. The co-expression network was visualized by Cytoscape software (version 3.8.2).

### Consensus Clustering Analysis

As distinct expression patterns shown in BECN1 and NF2 gene clusters, we sought to distribute IRI samples into several clusters by using the “ConsensusClusterPlus” R package based on the expression profile of the top 10 correlated genes (Wilkerson and Hayes, 2010). “Partition Around Medoids” algorithm was applied, and the Pearson distance was used to estimate similarity among samples. The resampling method was applied to sample 80% of patients for 50 times. All samples were clustered into k (2–8) groups. The optimal number of clusters was determined according to cumulative distribution function (CDF) and ∆(k). Moreover, the principal component analysis (PCA) was performed to analyze expression differences between clusters.

### Construction and Validation of DGF Predictive Signature

To identify DGF predictive signature, the least absolute shrinkage and selection operator (LASSO) regression analysis was performed to screen predictive markers among DFRGs using the “glmnet” package (Friedman et al., 2010; Simon et al., 2011; Tibshirani et al., 2012). A total of 203 IRI samples of GSE43974 were randomly divided into a training set and an internal testing set at a ratio of 1:1. The risk score of DGF predictive signature was calculated using coefficients ( β ) as follows: risk score = $\overset{n}{\underset{i=1}{\sum }}exp_{i}\times \beta i$ . The DGF risk score of each sample was calculated, and IRI samples were distributed into high-risk and low-risk groups based on the median risk score. Furthermore, GSE37838 was used as the validation set to test the robustness of the model. Expression levels of candidate genes in the signature were shown in the heatmap, and the patient distribution according to risk score was shown. The area under the receiver operating characteristic (AUC) was compared to evaluate the predictive efficiency.

### Estimation of Cell Abundance

xCell allows estimation of 64 immune and stromal cell infiltrations with validation using *in silico* simulations and cytometry immunophenotyping (Aran et al., 2017). The proportion of infiltrated cell was estimated by applying xCell to the data of GSE43974. The differences of infiltrated cells between high-risk and low-risk groups were analyzed, and cells with *p* value < 0.05 were found to be statistically significant.

### Mice and Renal IRI Model

All animal experiments were strictly conducted in accordance with the institutional animal care and use committee guidelines and approved by the Biomedical Ethics Committee of Sun Yat-sen University (Guangzhou, China). Male 8- to 10-week-old C57BL/6 mice (Guangdong Medical Experimental Animal Center, Guangzhou, China) were used in this research. Mice were anesthetized with an intraperitoneal injection of pentobarbital sodium (60 mg/kg), and the core body temperature was maintained between 34 and 36°C on a heating blanket. According to our previously reported method (Hu et al., 2021), a midline abdominal incision was taken, bilateral renal pedicles were blocked with non-traumatic vascular clips (FT222T, B.BRAUN, Germany) for 30 min, and then the clips were released to allow renal reperfusion for 24 h. The sham-operated mice only exposed bilateral renal pedicles without clamping. The mice were euthanized 24 h after surgery, and kidney tissues were harvested. Serum was collected for blood urea nitrogen (BUN) and serum creatinine detection using the mouse BUN ELISA Kit (MM-0692M2, MEIMIAN) and the mouse creatinine ELISA Kit (MM-0693M2, MEIMIAN).

### Cell Culture and Ferroptosis Induced by Erastin

Human proximal tubule epithelial cells (HK-2) were purchased from ATCC and validated by a short tandem repeat assay (STR) (IGE Biotech, Guangzhou, China). The cells were cultured in the DMEM/F12 medium (Gibco, CA, United States) supplemented with 10% fetal bovine serum (Gibco, CA, United States), 100 IU/ml penicillin, and 100 μg/ml streptomycin (Gibco, CA, United States) in an incubator containing 5% CO_2_ and 95% air at 37°C. For the induction of ferroptosis, HK-2 cells were treated with erastin (15 μM) for 24 h.

### Transfection of siRNA

DDIT3 siRNA and SLC2A3 siRNA were designed and synthesized from GenePharma (Shanghai, China). Before transfection, HK-2 cells were plated in a 6-well plate and incubated until 50–60% confluence. Cells were transfected for 24 h before treatment with ferroptosis inducer erastin, for another 24 h. Transfection mixture containing the Lipofectamine 2000 reagent (Invitrogen, Carlsbad, United States) and siRNA in an Opti-MEM medium (GIBCO, CA, United States) was added to the 6-well plate. siRNA-NC was served as the negative control. The siRNA sequences of target genes are shown in the Supplementary Table S1.

### Quantitative Real-Time PCR (qRT-PCR)

Total RNA was isolated from mouse kidney tissues using the TRIzol reagent (Invitrogen, Thermo Fisher Scientific, United States). Reverse transcription was performed using the 1st Strand cDNA Synthesis (+gDNA wiper) Kit (Vazyme, Nanjing, China). qRT-PCR was performed, according to the manufacturer’s protocol, using the ABI QuantStudioTM 5 (Thermo Fisher Scientific, Inc.) and SYBR Green PCR Master Mix (Vazyme, Nanjing, China). The relative mRNA expression levels were calculated using the 2^−ΔΔCt^ method with normalization to GAPDH mRNA. The primers used in this study are listed in Supplementary Table S2.

### Cell Viability Assay

After 6 h of siRNA transfection, HK-2 cells were plated in 96-well plates (5,000 cells per well) and incubated for 24 h. Then cells were treated with 15 μM of erastin for another 24 h. Cell viability was assessed by Cell Counting Kit-8 (CCK-8) Assay Kit (Vazyme, Nanjing, China). After treatment with erastin, 10 μl CCK-8 was added to each well and incubated at 37°C for 2 h. The optical density value of each well was measured with a microplate reader (Bio-Tek, United States) at 450 nm.

### Hematoxylin-Eosin, Immunohistochemical, and Immunofluorescent Staining

Kidney tissues from mice were embedded in paraffin and 4 μm sections were used for Hematoxylin-eosin (HE) staining to detect morphological changes.

To perform immunohistochemical (IHC) staining, paraffin-embedded kidney tissue was deparaffinized in xylene, rehydrated, and blocked with 10% goat serum for 30 min. Rabbit polyclonal anti-KIM-1 (2.5 ug/ml, Abcam, ab78494) primary antibodies were incubated at 4°C overnight and subsequently incubated with HRP-conjugated secondary antibody for IHC.

Frozen sections of mouse’s kidney were cooled to room temperature for 10 min and then blocked with 5% goat serum (Beyotime, Shanghai, China) at room temperature for 1 h. Sections were washed with phosphate-buffered saline (PBS) followed by incubation with primary antibodies against DDIT3 (1:200 dilution, CST, 2895T) and SLC2A3 (1:100 dilution, Santa Cruz, sc74399) overnight at 4°C. After washing for three times with PBS, fluorescent secondary antibodies were added and incubated at room temperature for 1 h. After counterstaining with DAPI (Beyotime, Shanghai, China) for 10 min, sections were sealed under glass coverslips with an anti-fading fluorescence medium (Applygen, Beijing, China). Images were captured with a fluorescent microscope (Nikon, Tokyo, Japan).

### Cellular ROS Detection

An intracellular ROS level was quantified by the ROS Assay Kit (Beyotime Biotechnology, Shanghai, China). HK-2 cells were plated on coverslips in a 6-well plate with a density of 4 × 10^5^ per well and cultured in the incubator containing 5% CO_2_ and 95% air at 37°C. After transfection and treatment by erastin, cells were incubated with 10 μM DCFH-DA at 37°C for 30 min in the dark and then washed with PBS. The fluorescence intensity was detected by the fluorescence microscope with an excitation of 488 nm and an emission of 525 nm.

### Iron Assay

After transfection and induction by erastin, HK-2 cells were washed with PBS and lysed for 2 h. Then the relative iron levels were assessed using the Iron Assay Kit (Applygen, Beijing, China), according to manufacturer’s protocol. The optical density was detected at the wavelength of 550 nm.

### Statistical Analysis

All statistical analyses were performed using SPSS 25.0 (SPSS Inc., Chicago, IL), GraphPad Prism software version 8 (GraphPad Software, San Diego, CA, United States), and R software 4.0.3. Cytoscape software (version 3.8.2) was used to visualize networks. Differences between two groups were compared using Student’s *t* test or the Mann–Whitney test. The association of clusters with DGF and donor types was analyzed using the Chi-square test. The qRT-PCR results were presented as mean ± standard error of mean. All the tests were two-sided, and a value of *p* < 0.05 was considered statistically significant.

## Results

### Identification and Functional Annotation of DFRGs in Renal IRI

**FIGURE 1:**
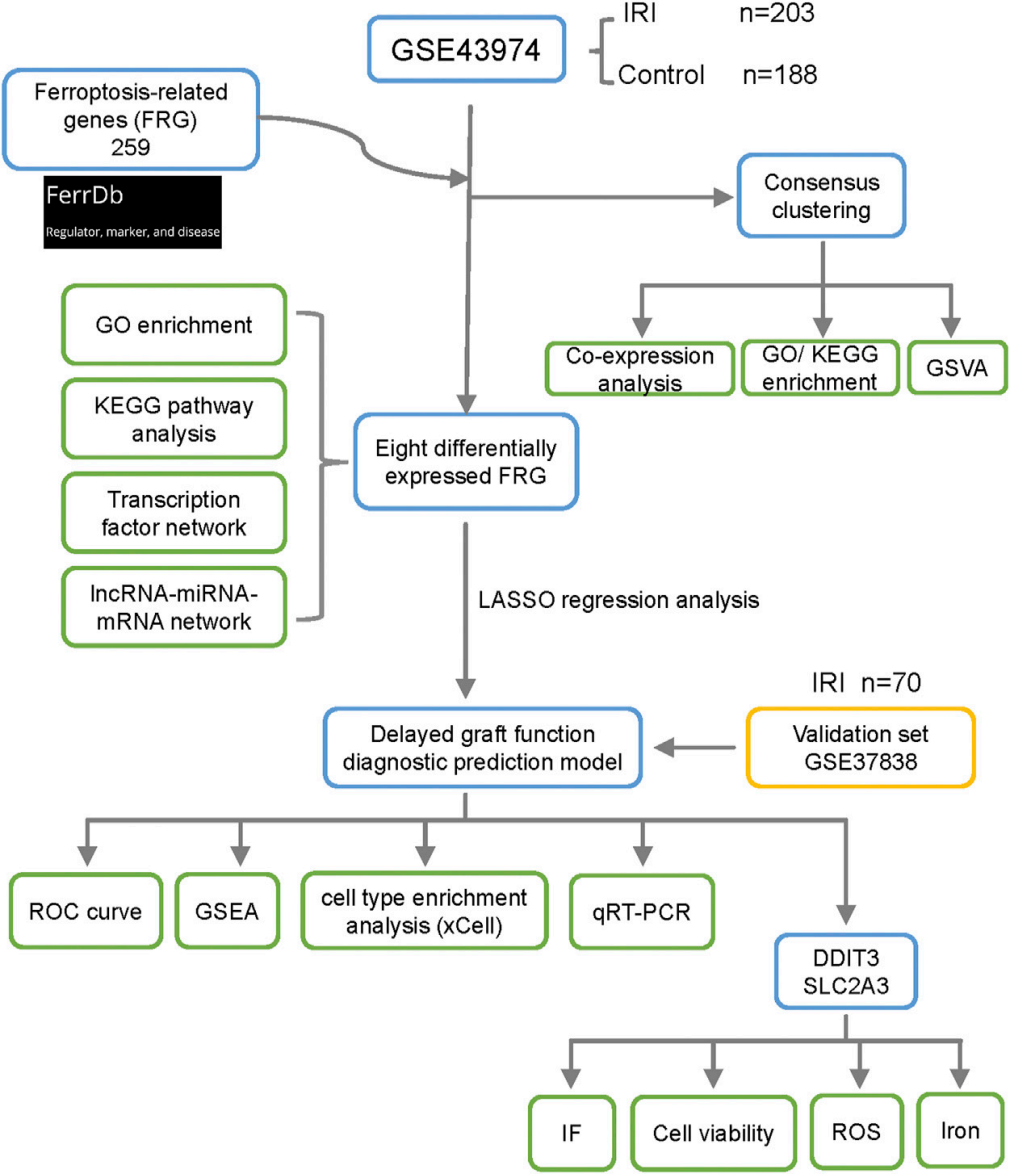
Flowchart of research design and analyzing process of this study. A differential expression analysis was performed between the IRI group and the control group in the GSE43974. FRGs were retrieved from the FerrDb database. A consensus clustering analysis was performed to identify ferroptosis-related clusters in IRI samples. A total of eight differentially expressed FRGs were selected for the functional enrichment analysis and network construction. Additionally, the LASSO regression analysis was performed to establish the delayed graft function predictive signature. The validation set GSE37838 with 70 IRI samples further verified the robustness of the signature. Cell infiltration of IRI samples was analyzed using the xCell algorithm. The mRNA expression of key genes was validated in the mouse IRI model by qRT-PCR. By analyzing cell viability, ROS generation, and relative iron level, knockdown of DDIT3 and SLC2A3 in HK-2 cell mitigated ferroptosis. FRG, ferroptosis-related gene; GO, gene ontology; KEGG, Kyoto Encyclopedia of Genes and Genomes; GSVA, gene set variation analysis; LASSO, least absolute shrinkage and selection operator; GSEA, gene set enrichment analysis; ROC, receiver operating characteristic; ROS, reactive oxygen species; IF, immunofluorescence.

**FIGURE 2:**
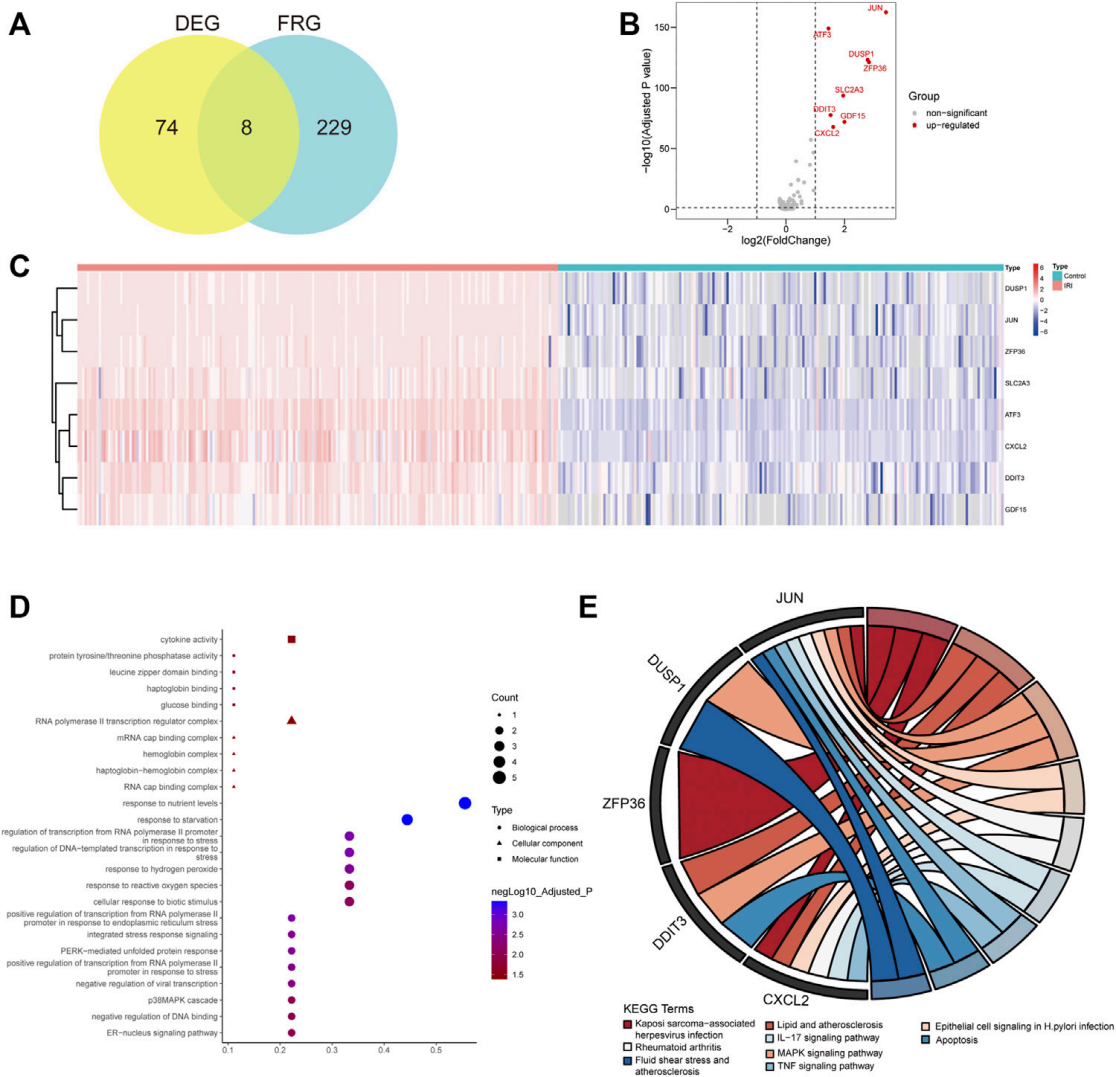
Differential expression analysis and functional enrichment analysis of FRGs. **(A)** Intersection between FRGs and differentially expressed genes (DEG) in renal IRI. **(B)** Volcano plot showing log2 (fold change) and adjusted P value of differentially expressed FRGs (DFRG). The gene symbols of DFRGs were labeled. **(C)** Heatmap showing the expression of DFRGs in the IRI group compared with the control group. **(D)** GO enrichment analysis of DFRGs in terms of biological process, cellular component, and molecular function. **(E)** KEGG pathway analysis of DFRGs. Terms with adjusted *p* < 0.05 were selected.

The flowchart of this study is shown in Figure 1. To identify DEGs in renal IRI, the GSE43974 geneset, which consisted of 203 kidney tissues obtained after reperfusion (IRI group) and 188 kidney tissues obtained before organ retrieval (control group), was used to perform the differential expression analysis. Then we identified 74 DEGs with | log(fold change) | ≥ 1 and FDR adjusted *p* < 0.05. A total of 259 FRGs were retrieved from the FerrDb database and 237 FRGs were listed in the microarray data of GES43974 (Supplementary Table S3). Common genes in both the DEGs and FRGs were defined as DFRGs. Thus, we identified eight DFRGs (ATF3, JUN, ZFP36, DUSP1, DDIT3, GDF15, CXCL2, and SLC2A3) which were all upregulated in IRI samples (Figures 2A–C; Supplementary Table S4). To gain an insight into the potentially involved function of DFRGs, we performed GO and KEGG pathway analyses. Results of the GO analysis showed that DFRGs were associated with hydrogen peroxide response, reactive oxygen species response, and regulation of transcription (Figure 2D). Additionally, KEGG results revealed that DFRGs were enriched in the MAPK signaling pathway, TNF signaling pathway, IL-17 signaling pathway, and apoptosis (Figure 2E).

### Construction of Transcription Factor Regulatory Network and ceRNA Network

**FIGURE 3:**
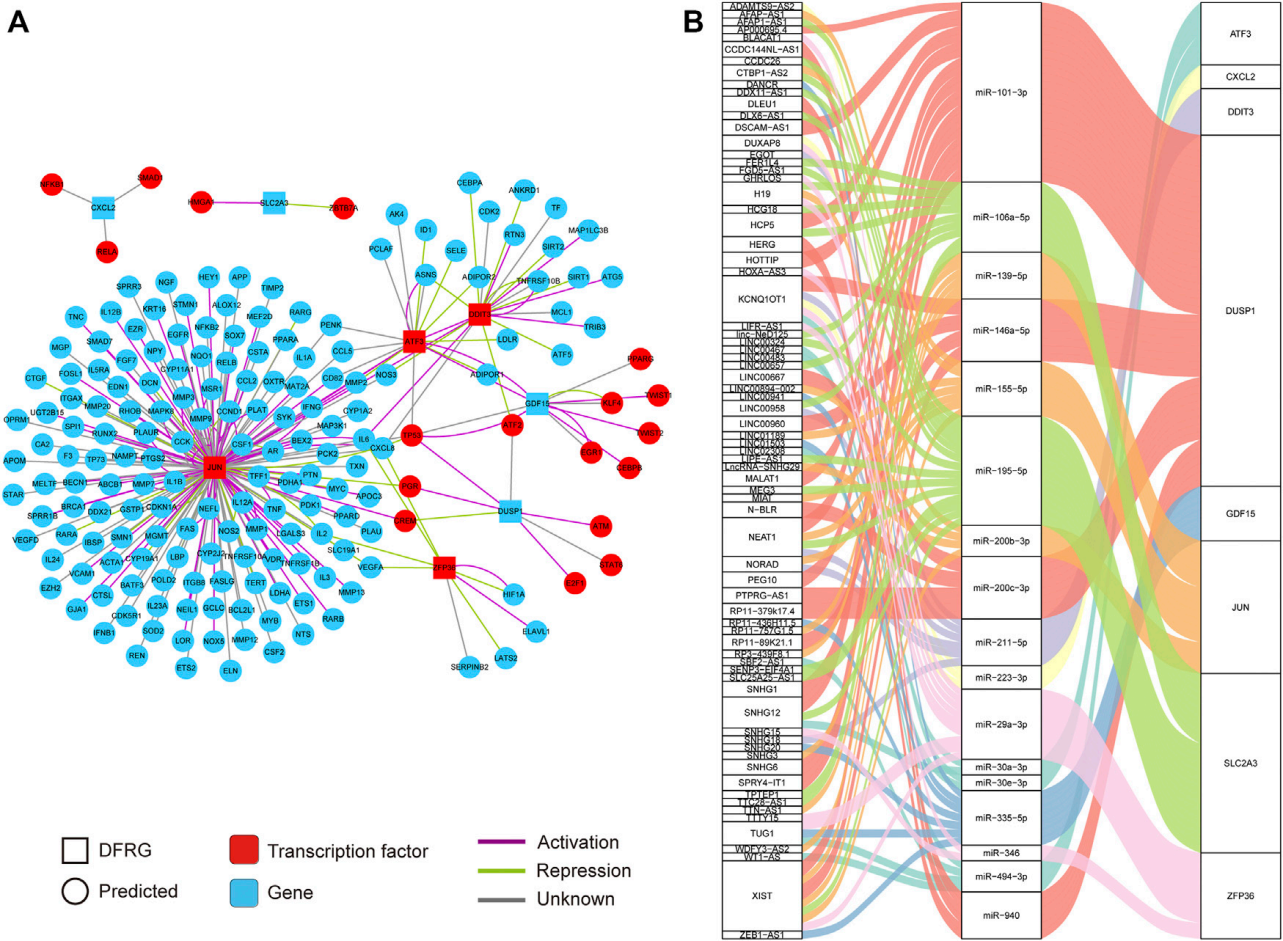
Construction of the transcription factor regulatory network and the lncRNA–miRNA–mRNA network based on eight DFRGs. **(A)** Transcription factor regulatory network of DFRGs constructed by using data from the TRRUST database. The interaction between transcription factors and targets were shown in green (repression) or purple lines (activation). **(B)** Construction of the lncRNA–miRNA–mRNA network with data from miRtarBase and LncACTdb databases.

Considering several transcription factors in DFRGs such as JUN and ZFP36, we attempted to investigate the transcription factor regulatory network of DFRGs. Dysregulation of transcription factors and their targets could lead to disease states; thus, elucidating the transcription factor–target interaction could help understand the regulatory relationship underlying the complex traits in IRI. As a database of transcription factor regulatory network based on experimental evidence, the TRRUST database was used to predict regulators or targets of DFRGs (Han et al., 2018). In the transcription factor regulatory network, transcription factors ATF3, JUN, ZFP36, and DDIT3 interacted with predicted targets, while CXCL2, SLC2A3, GDF15, and DUSP1 were targeted by several transcription factors (Figure 3A). We also showed evidence-based interactions (activation or repression) between transcription factors and targets. Additionally, a regulatory network of ceRNA could uncover crosstalk between protein-coding transcripts and non-coding transcripts and shed light on novel therapeutic targets (Tay et al., 2014). The miRtarBase and LncACTdb databases were used to identify experimentally validated miRNA and lncRNA of DFRGs (Figure 3B).

### Patient Stratification Based on Heterogeneously Expressed FRGs

**FIGURE 4:**
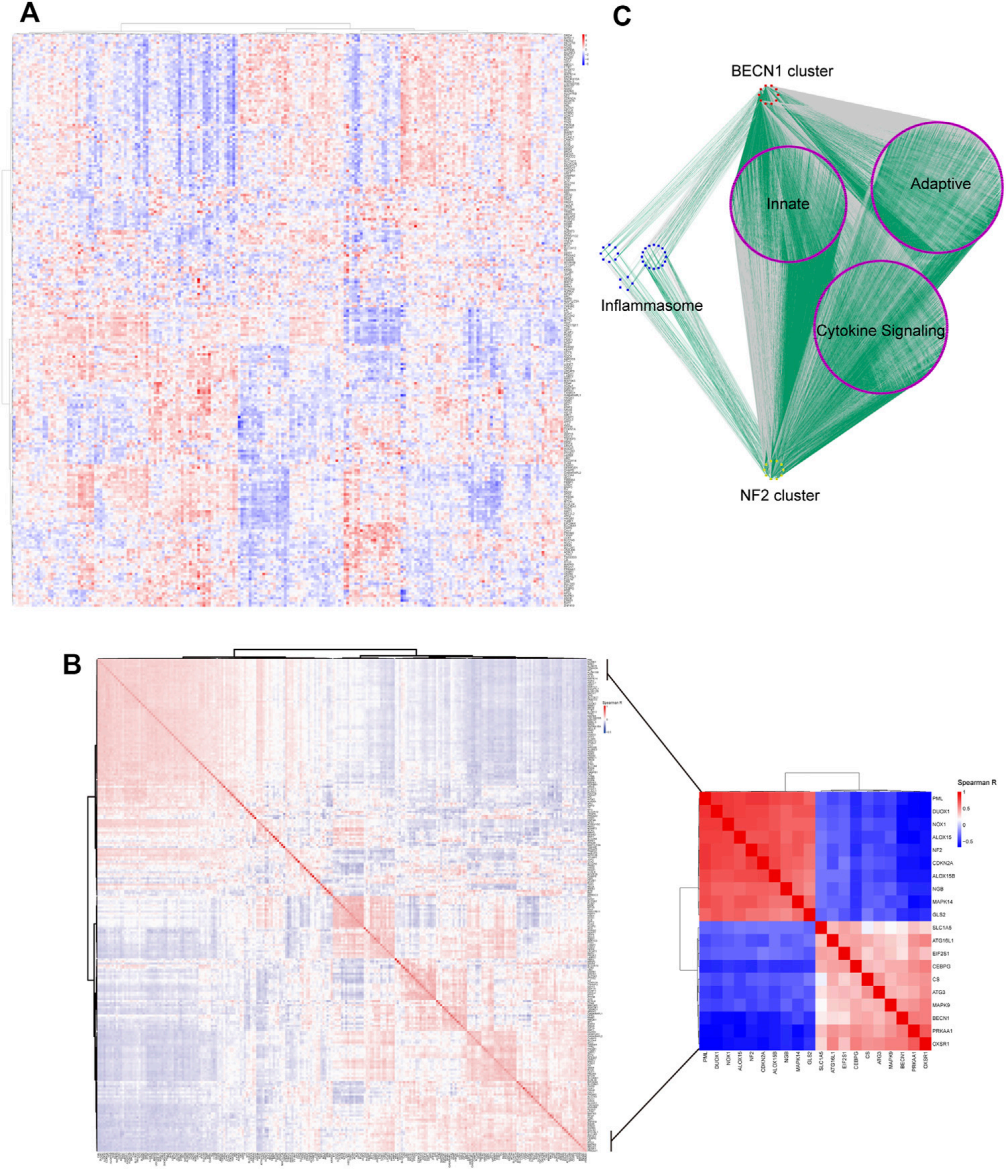
Two major clusters were identified in heterogeneously expressed FRGs. **(A)** Heatmap showing heterogeneously expressed FRGs across IRI samples. The color bar showed the expression level of genes. **(B)** Spearman correlation analysis revealed two major clusters with genes in one cluster opposite to the other cluster. The inset displayed the top 10 correlated genes in each cluster. The color bar showed the range of spearman correlation r. **(C)** Co-expression analysis of the top 10 correlated genes in each cluster with genes involved in inflammasome and immune pathways. Gray lines indicated negative correlation while green lines indicated positive correlation between genes.

As shown in the Figure 4A, 237 FRGs were heterogeneously expressed in the IRI samples and two distinct clusters were identified. Then we performed the Spearman correlation analysis using 237 FRGs to investigate their associations. Two clusters with positively co-expressed genes in one cluster but negatively co-expressed with the other were identified (Figure 4B; Supplementary Table S5). The top 10 correlated genes in one cluster included drivers of ferroptosis (DUOX1, NOX1, ALOX15, CDKN2A, ALOX15B, and MAPK14), suppressors (PML and NF2), and markers (NGB and MAPK14) (Figure 4B, inset). Wu et al. (2019) reported that inactivation of NF2 rendered cancer cells more sensitive to ferroptosis and highlighted its key role in prediction of responsiveness to ferroptosis-associated therapies. This cluster was hereafter referred as the NF2 cluster. On the other hand, the top 10 correlated genes in another cluster included drivers of ferroptosis (PRKAA1, BECN1, MAPK9, ATG3, CS, SLC1A5, and ATG16L1) and markers (OXSR1, CEBPG, and EIF2S1) (Figure 4B, inset). BECN1 had more interaction with other nine genes suggesting a potentially key role (Supplementary Figure S1), and thus we regarded this cluster as the BECN1 cluster.

It was noteworthy that the top 10 correlated genes in each cluster were co-expressed with genes in the inflammasome or immune-associated pathway which were retrieved from Benfeitas et al. (2019). As shown in Figure 4C, more immune- and inflammasome-related genes were positively co-expressed (green lines) with the NF2 cluster while negatively co-expressed (gray lines) with the BECN1 cluster (Supplementary Table S6). These results suggested a closer correlation of the NF2 cluster with immune infiltration and inflammation.

**FIGURE 5:**
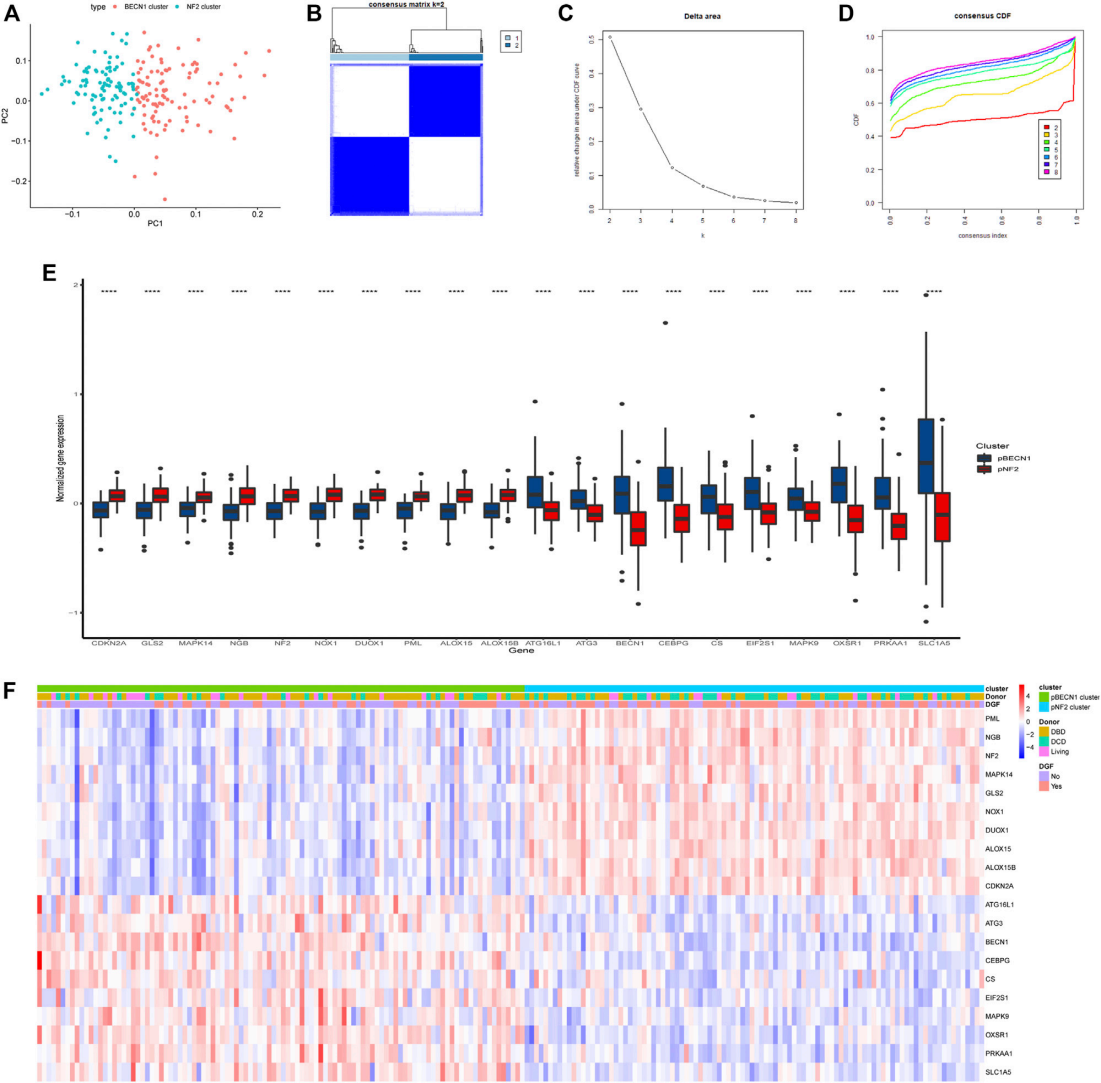
Consensus clustering analysis stratified patients into two clusters based on the top 10 correlated genes in the BECN1 gene cluster and the NF2 gene cluster. **(A)** Principal component analysis (PCA) showing distinct expression patterns of these two gene clusters. **(B)** Consensus matrix of IRI samples when k = 2. **(C,D)** Consensus CDF and relative change in the area under CDF curve when k = 2–8. **(E)** Gene expression level of top correlated genes in pBECN1 and pNF2 clusters. ****, *p* < 0.0001. **(F)** Heatmap showing association of the pBECN1 cluster and the pNF2 cluster with donor types and occurrence of DGF. The expression of the top 10 correlated genes in each cluster was shown. The color bar showed the expression level of genes. DBD, donation after brain death; DCD, donation after cardiac death.

The above results revealed opposite expression patterns of two gene clusters in IRI, which rendered us to investigate the possibility of patient stratification based on genes in BECN1 and NF2 gene clusters. The PCA showed distinct distribution patterns between the BECN1 cluster and the NF2 cluster (Figure 5A). The consensus clustering analysis was performed based on the top 10 correlated genes in the BECN1 cluster and the NF2 cluster. Obviously, k = 2 was selected as the optimal value with clustering increasing from k = 2 to k = 8, and the consensus matrix heatmap kept distinct boundaries when k = 2, suggesting robust clustering for all samples (Figures 5B–D). Therefore, IRI samples were divided into two clusters, namely, the pBECN1 cluster and the pNF2 cluster with high expression of top 10 genes in the BECN1 cluster and the NF2 cluster, respectively (Figure 5E). The association of clinical traits including occurrence of DGF and donor types with patient clusters was analyzed (Supplementary Table S7), and it revealed that DGF occurred more frequently in patients of the pNF2 cluster (*p* = 0.001). Additionally, the pNF2 cluster had more DCD patients, while the pBECN1 cluster had more DBD and living patients (*p* = 0.004; Figure 5F).

### Functional Enrichment Analysis of Clusters

**FIGURE 6:**
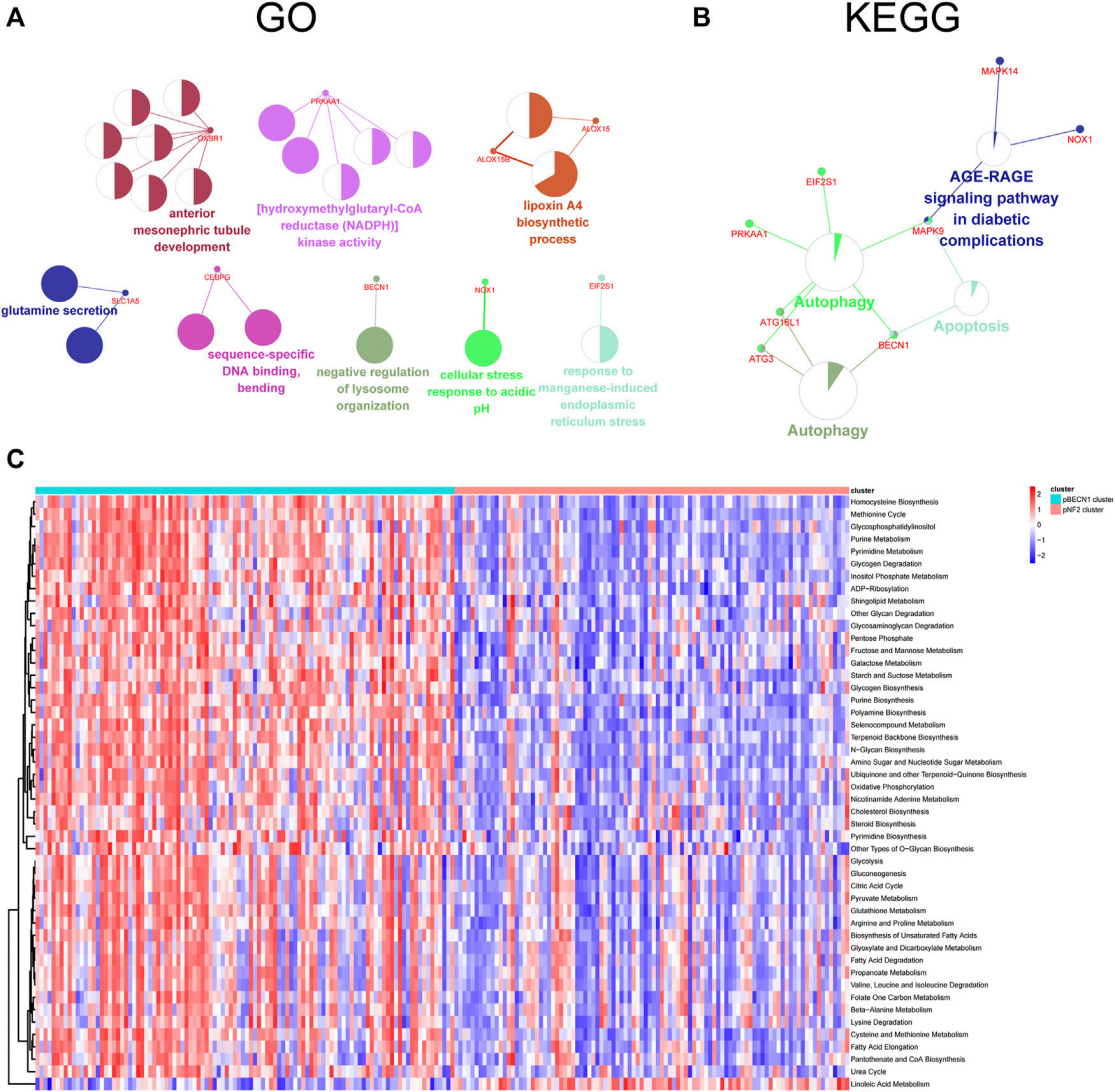
Functional enrichment analysis of the top 10 correlated genes and the pBECN1 cluster and the pNF2 cluster. **(A,B)** GO and KEGG enrichment analysis of the top 10 correlated genes in each cluster using the ClueGO plugin of Cytoscape software. **(C)** Gene Set Variation Analysis (GSVA) in terms of metabolic signaling signatures showing functional differences between the pBECN1 cluster and the pNF2 cluster. Terms with adjusted *p* < 0.05 were selected. The color bar showed the GSVA score in each sample.

To clarify the potential function of top 10 genes in each cluster, these genes were analyzed using the ClueGO plugin of Cytoscape software. The GO results showed that these genes were closely associated with metabolism including NADPH kinase activity, lipoxin synthesis, and glutamine secretion (Figure 6A). On the other hand, these genes were enriched in autophagy and apoptosis signaling pathways (Figure 6B). Additionally, the GSVA analysis in terms of the KEGG pathway revealed that regulation of actin cytoskeleton, mTOR signaling pathway, WNT signaling pathway, Notch signaling pathway, and P53 signaling pathway were significantly elevated across the pBECN1 cluster relative to the pNF2 cluster (adjusted *p* < 0.05; Supplementary Figure S2). Moreover, GSVA results in terms of metabolic pathways which were retrieved from Rosario et al. (2018) showed that the pBECN1 cluster had a higher score in most metabolic pathways, especially glutathione metabolism and fatty acid degradation pathways, which were closely associated with ferroptosis (Figure 6C; Supplementary Table S8). Meanwhile, the pNF2 cluster had a high GSVA score in only one pathway. These results suggested that the pBECN1 cluster was more metabolic active compared with the pNF2 cluster, suggesting more benefits from metabolic treatment in patients of the pBECN1 cluster.

### Construction and Validation of the DGF Predictive Signature

**FIGURE 7:**
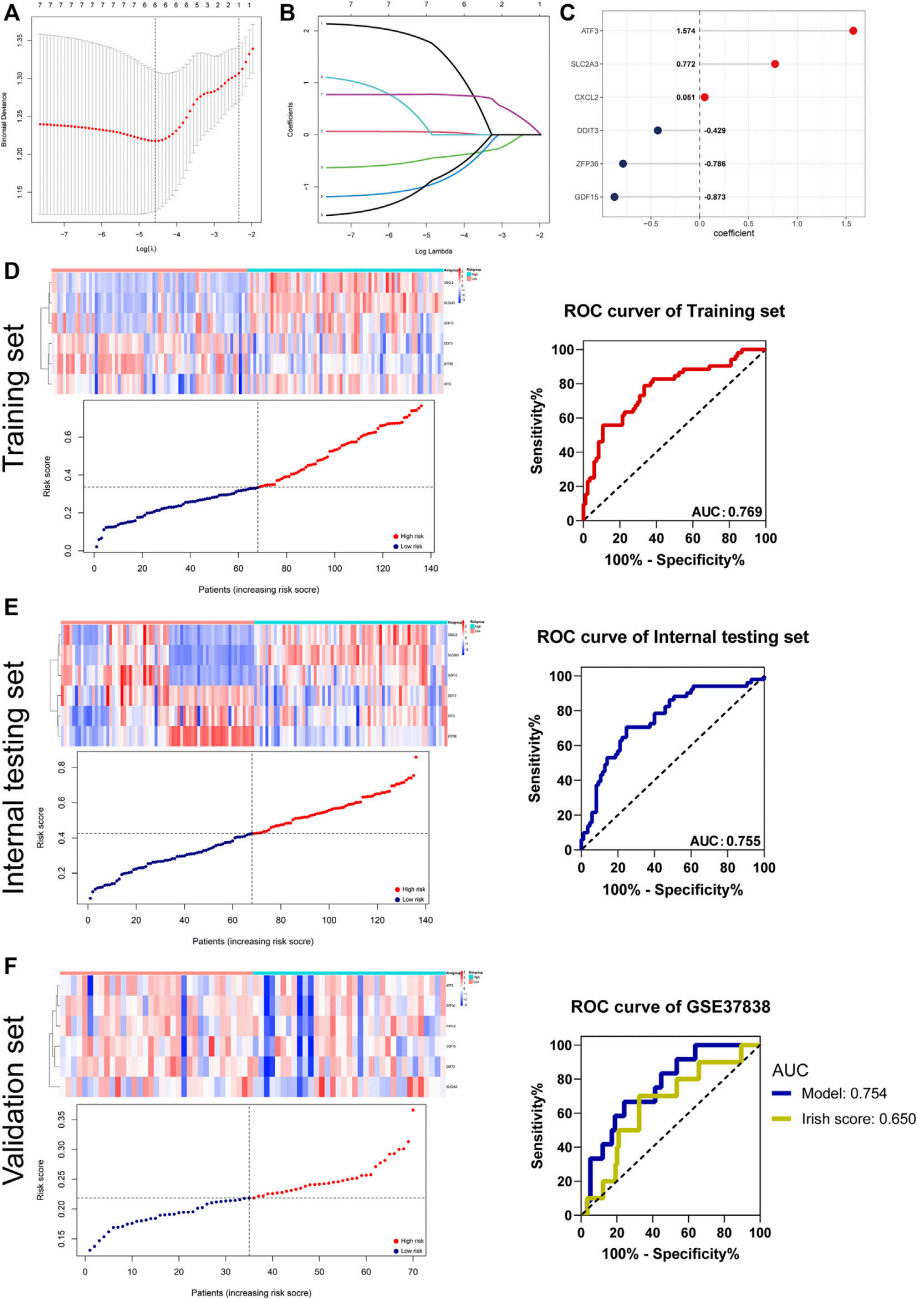
Establishment and validation of the DGF predictive signature. **(A,B)** DGF predictive signature was established from eight DFRGs. The lambda.min was selected as the best lambda value of the model. **(C)** LASSO coefficients of candidate genes in the signature. **(D–F)** Evaluating the performance of the signature in the training set, internal testing set, and validation set (GSE37838). The heatmaps showing gene expression of six genes (CXCL2, SLC2A3, GDF15, DDIT3, ZFP36, and ATF3) in the signature. Patients’ distribution based on the median risk score. ROC curves presenting the efficacy of the signature for predicting DGF in IRI patients. Area under the ROC curve (AUC) was calculated and compared.

The DGF is mainly a consequence of renal IRI after the kidney transplantation, which may lead to rejection and worse allograft survival (Siedlecki et al., 2011). As aforementioned results revealed close association between ferroptosis and DGF, we attempted to construct a robust predictive signature for DGF based on DFRGs. To this end, IRI samples of GSE43974 were randomly distributed into the training set or the internal testing set (1:1). The LASSO regression analysis screened ATF3, SLC2A3, CXCL2, DDIT3, ZFP36, and GDF15 as candidate genes of the DGF predictive model, with the coefficients of 1.574, 0.772, 0.051, −0.429, −0.786, and −0.873, respectively (Figures 7A–C). The risk score of each patient was calculated, and patients were divided into high-risk or low-risk groups based on the median risk score. Subsequently, the receiver operating characteristic (ROC) curves were used to evaluate predictive efficiency of the signature. The AUCs of ROC curves in the training set and internal testing set were 0.769 and 0.755 (Figures 7D,E). To further validate the robustness, the GSE37838 geneset comprising 70 IRI samples was used as the validation set. The AUC in the validation set was 0.754 and outperformed the Irish score (AUC 0.65), which was a DGF-predicting nomogram composed of 16 donor’s and recipient’s risk factors (Salvadori and Tsalouchos, 2019) (Figure 7F). Collectively, we constructed a ferroptosis-related DGF predictive signature in patients with renal IRI, and this signature outperformed the traditional Irish score.

### Functional Annotation and Correlation of the Signature With Ferroptosis

**FIGURE 8:**
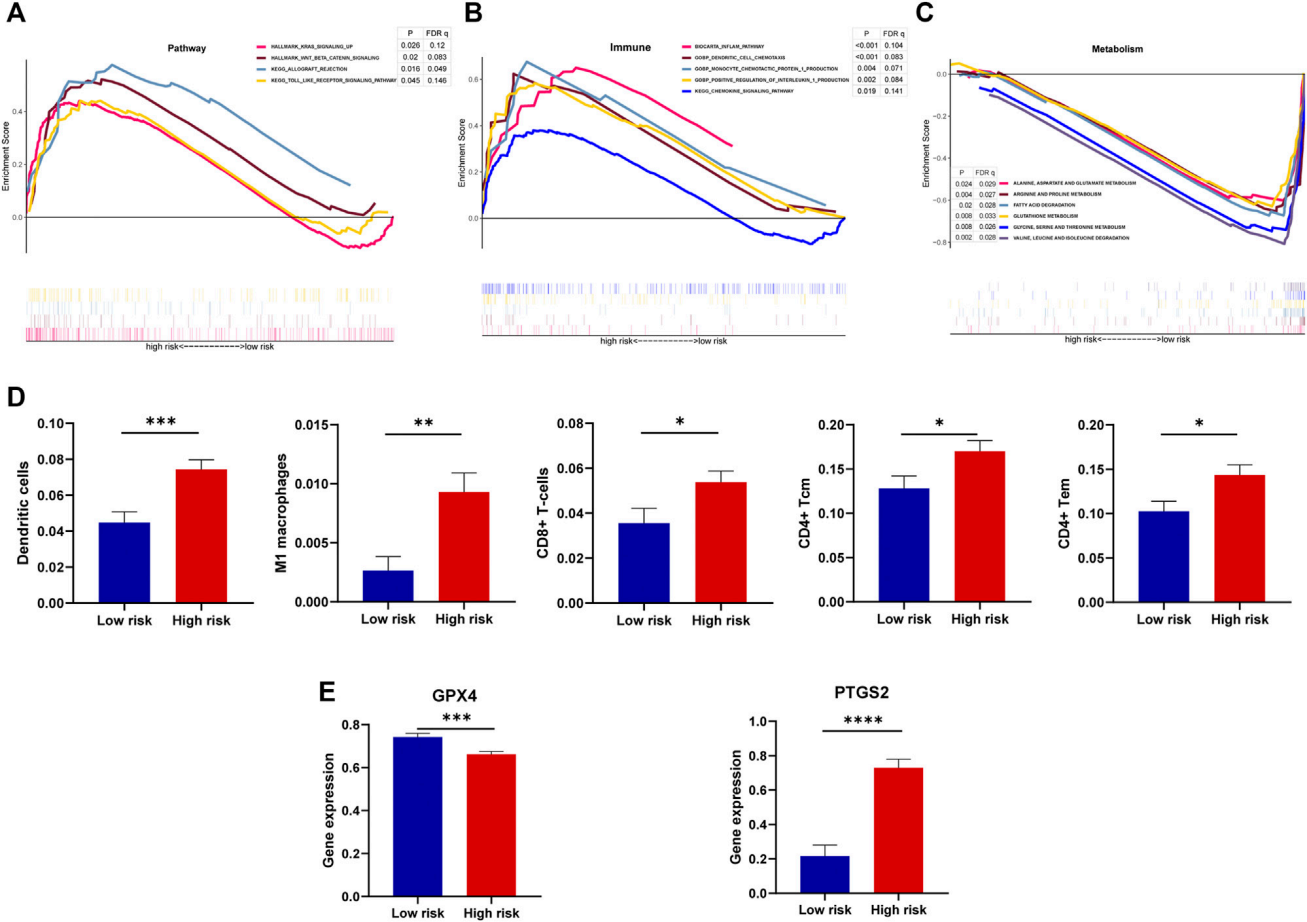
Association of the model with involved function, immune cell infiltration, and ferroptosis. **(A–C)** GSEA showing the involved function of high-risk and low-risk groups in terms of the DGF-related pathway, immune, and metabolism. Terms with *p* < 0.05 and FDR q < 0.25 were considered statistically significant. **(D)** Differences of immune cell infiltration between low-risk and high-risk groups, estimated by the xCell algorithm. **(E)** Gene expression of key ferroptosis markers, namely, GPX4 and PTGS2 between risk groups. *, *p* < 0.05; **, *p* < 0.01; ***, *p* < 0.001; ****, *p* < 0.0001.

Investigating involved function or signaling pathways of high-risk or low-risk groups helped identify signaling pathways related to DGF and seek possible targeted treatment. The results of GSEA showed that high-risk samples were enriched in the KRAS signaling pathway, WNT/β-catenin signaling pathway, Toll-like receptor signaling, and allograft rejection (Figure 8A). In terms of immune and inflammation, dendritic cells and the chemokine-related pathway were significantly enriched in the high-risk group (Figure 8B). Additionally, the low-risk group was associated with metabolism including glutathione and fatty acid metabolism (Figure 8C).

During the progression of DGF, macrophages may stimulate chemokines production by dendritic cells and subsequently activate T lymphocytes (Siedlecki et al., 2011). To clarify the changes of immune cells, the xCell algorithm was performed to estimate cell infiltration of each sample in IRI (Aran et al., 2017). The results showed that samples with high risks of DGF were infiltrated with dendritic cells, M1 macrophages, CD8^+^ T cells, CD4^+^ T central memory cells, and CD4^+^ T effector memory cells (Figure 8D). Moreover, the vital regulator glutathione peroxidase 4 (GPX4), which prevented iron-dependent formation of lipid ROS, was decreased in ferroptosis (Friedmann Angeli et al., 2014), whereas prostaglandin-endoperoxide synthase 2 (PTGS2) were increased. Intriguingly, the high-risk group showed decreased GPX4 and increased PTGS2 compared with the low-risk group (Figure 8E), suggesting that high-risk patients may present severe ferroptosis. Therefore, these results not only indicated close correlation of the signature with ferroptosis but also reflected the changes of immune cells in DGF.

### Validation of Gene Expression and Function of DDIT3 and SLC2A3 in Ferroptosis

**FIGURE 9:**
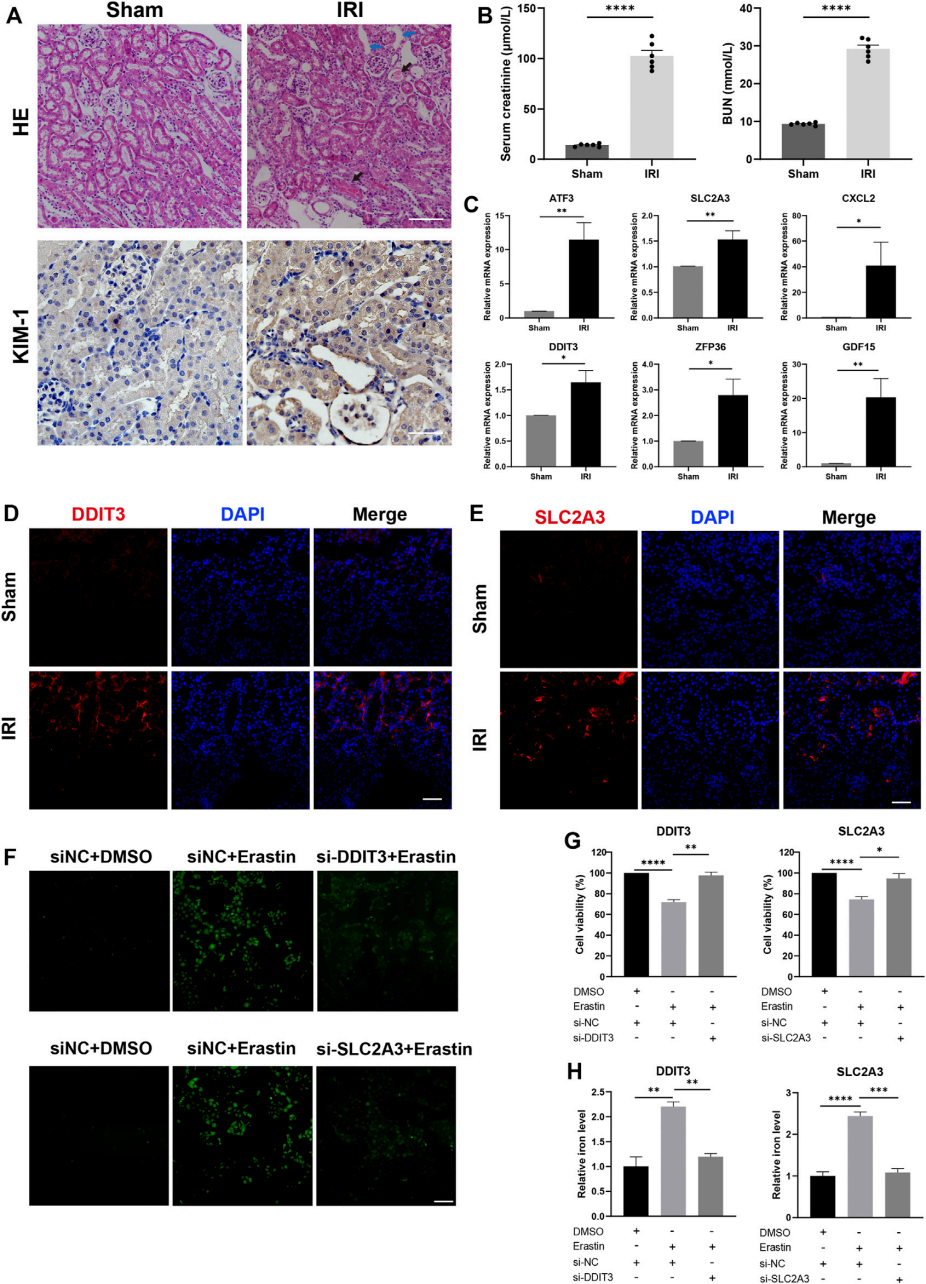
Expression of candidate genes in the renal IRI mouse model and knockdown of DDIT3 and SLC2A3 in HK-2 cell alleviated ferroptosis. **(A)** Representative images of hematoxylin and eosin staining and immunohistochemistry staining of KIM-1 in IRI mice and sham mice. The image of IRI showed tubular cell death, effacement of brush border (blue arrow), and proteinaceous casts in tubules (black arrow). Scale bars, 100 μm. *n* = 3 per group. **(B)** Serum creatinine and BUN were measured in the IRI group and the sham group. *n* = 6 per group. **(C)** mRNA expression level of ATF3, SLC2A3, CXCL2, DDIT3, ZFP36, and GDF15 in kidney tissue of the IRI group and the sham group. *n* = 3 per group. **(D,E)** Representative immunofluorescence images of DDIT3 and SLC2A3 in kidney tissue of IRI mice and sham mice. Nuclei were stained with DAPI (blue). Scale bars, 100 μm. *n* = 3 per group. **(F)** ROS generation was detected using DCFH-DA staining. HK-2 cells were transfected with si-DDIT3, si-SLC2A3, or si-NC and treated with erastin (15 μM, 24 h) or DMSO. Scale bars, 200 μm. **(G)** Cell viability was measured by CCK-8 assay. *n* = 3 per group. **(H)** Relative iron level in HK-2 cell was measured. Data were presented as the mean ± standard error of mean. *p < 0.05, **p < 0.01, ***p < 0.001, ****p < 0.0001. IRI, ischemia-reperfusion injury; BUN, blood urea nitrogen; NC, negative control; CCK-8, Cell Counting Kit-8.

To further validate the expression of six genes in the renal IRI, we first constructed a mouse IRI model (ischemia for 30 min and reperfusion for 24 h). The HE staining of IRI showed tubular cell death, increased inflammatory cell infiltration, effacement of brush border, and proteinaceous casts in the tubule compared with the sham group (Figure 9A). Immunohistochemistry demonstrated strong KIM-1 staining in IRI, a typical biomarker for renal proximal tubule injury (Figure 9A). Additionally, serum creatinine and BUN were significantly increased in IRI compared with the sham group (Figure 9B). The mRNA expression of six genes was verified by qRT-PCR in mouse kidney tissues after IRI. Results of qRT-PCR showed that six genes, namely, ATF3, SLC2A3, CXCL2, DDIT3, ZFP36, and GDF15, were significantly upregulated in the mice IRI group compared with the sham group (*p* < 0.05; Figure 9C).

As few studies analyzed DDIT3 and SLC2A3 in renal IRI, we further investigated their correlation with ferroptosis in IRI. Immunofluorescence staining of kidney tissue showed stronger staining of DDIT3 and SLC2A3 in IRI mice (Figures 9D,E). To investigate whether knockdown of DDIT3 and SLC2A3 could alleviate ferroptosis, we transfected HK-2 cells with siRNAs targeting DDIT3 or SLC2A3, and cells were treated by a ferroptosis inducer erastin (15 μM) for 24 h. ROS generation was increased in ferroptosis, and knockdown of DDIT3 and SLC2A3 mitigated increased ROS generation caused by ferroptosis (Figure 9F). The CCK-8 assay showed that cell viability decreased significantly after treatment of erastin, and knockdown of DDIT3 or SLC2A3 alleviated cell death caused by erastin (Figure 9G). Moreover, increased relative iron levels in ferroptosis were mitigated by knockdown of DDIT3 or SLC2A3 (Figure 9H). Collectively, consistent with the analysis of GEO data, six model genes were upregulated in IRI. Interference of DDIT3 or SLC2A3 expression inhibited ferroptosis induced by erastin.

## Discussion

Renal IRI occurring in kidney transplantation usually leads to AKI, DGF, and eventually graft loss. Much progress has been obtained in understanding the underlying mechanism and developing therapeutic methods in renal IRI but it is still far from satisfactory. Ferroptosis is an iron-dependent type of programmed cell death and contributes to tubular cell death in renal IRI (Linkermann et al., 2014). The present study attempted to reveal the characterization of ferroptosis in renal IRI. We identified two gene clusters with heterogeneously expressed FRGs and stratified patients into two clusters (pBECN1 and pNF2). Cluster pBECN1 was regarded as metabolic active subtype and tended to have less DGF. Inversely, cluster pNF2 was metabolic exhausted with higher incidence of DGF. Additionally, a DGF predictive signature consisting of six DFRGs (ATF3, SLC2A3, CXCL2, DDIT3, ZFP36, and GDF15) was constructed and validated. Furthermore, we verified the expression of six DFRGs in IRI and revealed that knockdown of DDIT3 or SCL2A3 alleviated ferroptosis in renal IRI.

Ferroptosis is an iron-dependent necrotic type of regulated cell death, characterized by accumulation of lipid peroxide which is regulated by GPX4 (Dixon et al., 2012). Failure of lipid peroxide to be metabolized by the GPX4-catalyzed reduction and massive production of ROS results in ferroptosis (Friedmann Angeli et al., 2014). The relevant role of ferroptosis in renal IRI had been uncovered by Linkermann et al. (2014). In addition, it was reported that combination therapy targeting necroptosis and ferroptosis might be effective in preventing renal IRI (Muller et al., 2017). We found all DFRGs upregulated in renal IRI and significantly enriched in inflammatory signaling pathways such as the MAPK signaling pathway, IL-17 signaling pathway, and TNF signaling pathway. It was reported that the MAPK signaling pathway played a role in ferroptosis by affecting ROS production (Nakamura et al., 2019). As several genes in DFRGs, such as JUN, ZFP36, DDIT3, and ATF3, were transcription factors, the TRRUST database was used to construct a transcription factor regulatory network with all DFRGs and uncover evidence-based interactions between transcription factors and targets (Han et al., 2018). Among DFRGs, six genes, namely, ATF3, SLC2A3, CXCL2, DDIT3, ZFP36, and GDF15, were selected as candidate genes of the DGF predictive model. The qRT-PCR results showed that these genes were upregulated in mouse IRI kidney tissue. ATF3 was reported to promote ferroptosis by suppressing system Xc^−^ (Wang et al., 2020), and inhibition of ATF3 alleviated AKI by suppressing ferroptosis (Wang et al., 2021). Additionally, CXCL2 was involved in a ferroptosis-associated signature for predicting graft loss after kidney transplantation (Fan et al., 2021). It was reported that RNA-binding protein ZFP36 suppressed ferroptosis by regulating autophagy and was identified as the therapeutic target of liver fibrosis (Zhang et al., 2020). Knockdown of GDF15 promoted ferroptosis by interfering SLC7A11 and the system Xc^−^ function (Chen et al., 2020), and it was upregulated in renal IRI though deficiency of it exacerbated kidney injury (Liu et al., 2020). DDIT3 was involved in the interaction between ferroptosis and apoptosis (Lee et al., 2018). But few studies investigated the roles of DDIT3 and SLC2A3 in renal IRI. The immunofluorescence staining showed that SLC2A3 and DDIT3 were highly expressed in kidney tissue from the IRI group compared with the sham group. Furthermore, knockdown of SLC2A3 and DDIT3 significantly rescued decreased cell viability, increased iron level, and increased ROS caused by ferroptosis. These results verified the upregulation of six candidate genes in renal IRI tissue and uncovered the role of SLC2A3 and DDIT3 in promoting ferroptosis.

Consensus clustering is an unsupervised approach to identify subtypes based on gene expression profiles (Wilkerson and Hayes, 2010). It has been applied to identify subtypes in clear cell renal cell carcinoma and hepatocellular carcinoma based on expression profiles of FRGs (Bai et al., 2021; Liu et al., 2021). As shown in Figure 4, two clusters of heterogeneously expressed FRGs in IRI were formed. The top 10 correlated genes in one cluster included NF2, which was proved to play a pivotal role in ferroptosis (Wu et al., 2019). Hence, this cluster was defined as the NF2 cluster. Among the top 10 correlated genes in another cluster, BECN1 was a driver of ferroptosis (Song et al., 2018), and it interacted with most of the other genes. It suggested that BECN1 might play a key role among these genes, and thus we regarded this cluster as the BECN1 cluster. Moreover, IRI usually elicited an adaptive immune response characterized by activation of T cells and subsequent T cell–mediated injury (Day et al., 2006). Inflammasomes responded to injury and resulted in production of pro-inflammatory cytokines (Schroppel and Legendre, 2014). A co-expression analysis revealed close association of the NF2 cluster with immune- and inflammasome-related genes. This finding may suggest that the NF2 cluster was associated with more severe kidney injury. Considering the opposite expression patterns between BECN1 and NF2 clusters, the consensus clustering analysis was performed to successfully divided patients into two distinct clusters (the pBECN1 cluster and the pNF2 cluster) based on the top 10 correlated genes in BECN1 and NF2 clusters. The GSVA analysis showed that the pBECN1 cluster had higher GSVA scores in most metabolic signatures, and thus the pBECN1 cluster was metabolically active. Inversely, pNF2 had low GSVA scores in most metabolic signatures, and we regarded pNF2 as a metabolically exhausted cluster. Cluster pNF2 had higher incidence of DGF, indicating close association of ferroptosis with DGF. These results indicated possible responses of IRI patients to metabolic therapeutics.

DGF usually results from renal IRI, and it is a challenge for allograft survival after kidney transplantation (Schroppel and Legendre, 2014). Considering close association of clusters with DGF, we constructed a ferroptosis-related model to predict occurrence of DGF. The model exhibited robustness in predicting DGF in the training set, internal testing set, and validation set. The predictive efficacy of this model was better than Irish score, which was a nomogram combining donor’s and recipient’s risk factors for DGF prediction (Irish et al., 2010). Additionally, injured cells release inflammatory cytokines such as IL-1α, TNFα and subsequently activated T cells and macrophages during IRI (Li and Okusa, 2006). Our results revealed more infiltration of dendritic cell, M1 macrophages, CD8^+^ T cells, CD4^+^ T central memory cells, and CD4^+^ T effector memory cells in high-risk patients. These results suggested that patients with high risks of DGF may experience more infiltration of inflammation and even more severe IRI. Ferroptosis was characterized by increased PTGS2 and decreased expression of GPX4. Consistently, the high-risk group had lower expression of GPX4 but over-expressed PTGS2. Collectively, it may suggest more severe ferroptosis in the high-risk group and possible benefits from treatment targeting ferroptosis for high-risk patients.

However, some limitations of this research should be noted. First, more datasets were needed to further verify the robustness of the classification and predictive signature. Secondly, clinical samples could be used to validate the gene expression. More research should be carried out to investigate regulatory mechanism of DDIT3 and SLC2A3 in ferroptosis and renal IRI.

## Conclusion

In this study, we first identified vital DFRGs in renal IRI and constructed a transcription factor regulatory network and a lncRNA–miRNA–mRNA network based on DFRGs. Additionally, we stratified IRI patients into the pBECN1 cluster and the pNF2 cluster based on two gene clusters with distinct expression patterns. Cluster pBECN1 had less DGF and was regarded as the metabolic active subtype, suggesting possible response to metabolic therapeutics. Inversely, the pNF2 cluster was metabolic exhausted and had higher incidence of DGF. Furthermore, we constructed and validated a ferroptosis-associated DGF predictive signature with robust efficacy. Knockdown of DDIT3 and SLC2A3 could alleviate ferroptosis. Collectively, these results provided an insight into characterization of ferroptosis in renal IRI, though more research and samples were needed.

## Data Availability

The datasets presented in this study can be found in online repositories. The names of the repository/repositories and accession number(s) can be found in the article.
