# Supplementary material for: Identification of Subtypes and a Delayed Graft Function Predictive Signature Based on Ferroptosis in Renal Ischemia-Reperfusion Injury
Source: Front Cell Dev Biol. 2022 Feb 8;10:800650. doi: 10.3389/fcell.2022.800650 (PMC8861527; doi:10.3389/fcell.2022.800650)

Supplementary material

| Supplementary Table S1: siRNA sequences for DDIT3 and SLC2A3 | | | |
| --- | --- | --- | --- |
| siRNA | Species |  | Sequence |
| si-DDIT3 | Homo sapiens | Sense | GCAUCGUUGUUGGAAUUCUTT |
|  |  | Antisense | AGAAUUCCAACAACGAUGCTT |
| si-SLC2A3 | Homo sapiens | Sense | GCGCAUGAAGGAGAAAGAATT |
|  |  | Antisense | UUCUUUCUCCUUCAUGCGCTT |
| si-NC | Homo sapiens | Sense | UUCUCCGAACGUGUCACGUTT |
|  |  | Antisense | ACGUGACACGUUCGGAGAATT |

| Supplementary Table S2: Primer sequences for qRT-PCR | | | |
| --- | --- | --- | --- |
| Primer | Species |  | Sequence |
| ATF3 | Mus musculus | Forward | GAGGATTTTGCTAACCTGACACC |
|  |  | Reverse | TTGACGGTAACTGACTCCAGC |
| SLC2A3 | Mus musculus | Forward | ATGGGGACAACGAAGGTGAC |
|  |  | Reverse | GTCTCAGGTGCATTGATGACTC |
| CXCL2 | Mus musculus | Forward | CCAACCACCAGGCTACAGG |
|  |  | Reverse | GCGTCACACTCAAGCTCTG |
| DDIT3 | Mus musculus | Forward | CTGGAAGCCTGGTATGAGGAT |
|  |  | Reverse | CAGGGTCAAGAGTAGTGAAGGT |
| ZFP36 | Mus musculus | Forward | CCGAATCCCTCGGAGGACTT |
|  |  | Reverse | GAGCCAAAGGTGCAAAACCA |
| GDF15 | Mus musculus | Forward | CTGGCAATGCCTGAACAACG |
|  |  | Reverse | GGTCGGGACTTGGTTCTGAG |
| GAPDH | Mus musculus | Forward | AATGGTGAAGGTCGGTGT |
|  |  | Reverse | GTGGAGTCATACTGGAACATGTAG |

Supplementary Table S3: The list of 237 FRGs in GSE43974 dataset

| **FRG** | **logFC** | **adj.P.Val** | **FRG** | **logFC** | **adj.P.Val** |
| --- | --- | --- | --- | --- | --- |
| RPL8 | -0.17785 | 6.59E-08 | FTMT | 0.011791 | 0.750926 |
| IREB2 | -0.03724 | 0.123873 | HSPA5 | 0.23295 | 8.58E-05 |
| CS | -0.10041 | 0.00013 | ATF4 | 0.269734 | 3.15E-12 |
| ACSF2 | 0.172657 | 0.33554 | HELLS | 0.02955 | 0.098223 |
| NOX1 | 0.009344 | 0.752114 | SCD | -0.12555 | 0.388213 |
| CYBB | -0.0455 | 0.101744 | FADS2 | -0.00432 | 0.90404 |
| NOX3 | 0.010159 | 0.780097 | SRC | 0.012501 | 0.527529 |
| NOX4 | 0.166529 | 0.378801 | STAT3 | 0.026897 | 0.805443 |
| NOX5 | 0.010243 | 0.770217 | PML | 0.010904 | 0.648278 |
| DUOX1 | 0.016218 | 0.507779 | NFS1 | -0.00074 | 0.977585 |
| DUOX2 | 0.024168 | 0.329372 | TP63 | 0.007939 | 0.793542 |
| G6PD | 0.015231 | 0.567822 | CDKN1A | 0.851341 | 6.11E-58 |
| PGD | -0.10963 | 0.01226 | MIR137 | 0.019225 | 0.499177 |
| VDAC2 | 0.003636 | 0.916904 | ENPP2 | -0.00026 | 0.998269 |
| PIK3CA | 0.001585 | 0.948584 | FH | -0.2034 | 0.015545 |
| FLT3 | 0.000466 | 0.989646 | CISD2 | -0.09179 | 0.03406 |
| SCP2 | 0.02004 | 0.631355 | MIR9-1 | 0.010465 | 0.769221 |
| TP53 | -0.02769 | 0.256145 | MIR9-3 | 0.008721 | 0.80913 |
| ACSL4 | 0.015388 | 0.58466 | CBS | -0.12034 | 0.004589 |
| LPCAT3 | 0.043771 | 0.64639 | ISCU | -0.17392 | 3.27E-06 |
| NRAS | -0.00342 | 0.942879 | ACSL3 | 0.017422 | 0.692463 |
| KRAS | -0.00884 | 0.69265 | OTUB1 | -0.03018 | 0.045163 |
| HRAS | -0.05719 | 0.00012 | CD44 | -0.03839 | 0.50998 |
| TF | -0.07286 | 0.210745 | BRD4 | 0.012758 | 0.549587 |
| TFRC | 0.088923 | 0.53768 | PRDX6 | -0.16445 | 0.010132 |
| TFR2 | 0.009762 | 0.795918 | SESN2 | 0.126764 | 2.03E-09 |
| SLC38A1 | -0.11945 | 0.004871 | NF2 | 0.007044 | 0.783804 |
| SLC1A5 | 0.102529 | 0.184422 | ARNTL | -0.01062 | 0.752206 |
| GLS2 | 0.007732 | 0.785425 | JUN | 3.41904 | 5.4E-163 |
| GOT1 | -0.0899 | 0.253852 | CA9 | 0.011411 | 0.69012 |
| ALOX5 | -0.00432 | 0.891799 | TMBIM4 | -0.21577 | 2.36E-09 |
| KEAP1 | -0.05475 | 0.002907 | PLIN2 | 0.363178 | 0.000347 |
| HMOX1 | 0.527915 | 1.95E-05 | MIR212 | -0.00164 | 0.965524 |
| ATG5 | -0.07929 | 0.004471 | AIFM2 | -0.00803 | 0.76061 |
| ATG7 | -0.02204 | 0.541824 | LAMP2 | -0.04353 | 0.608491 |
| NCOA4 | -0.10408 | 0.022891 | ZFP36 | 2.838409 | 6.7E-122 |
| ALOX12 | 0.012915 | 0.691504 | PROM2 | -0.13864 | 0.17071 |
| ALOX12B | 0.008083 | 0.795731 | CHMP5 | -0.22848 | 1.04E-07 |
| ALOX15 | 0.016805 | 0.524377 | CHMP6 | -0.01399 | 0.514045 |
| ALOX15B | 0.00184 | 0.952435 | CAV1 | -0.20926 | 4.26E-06 |
| ALOXE3 | 0.016768 | 0.601158 | GCH1 | 0.049763 | 0.124739 |
| PHKG2 | -0.0314 | 0.259537 | PTGS2 | 0.470617 | 3.78E-11 |
| ACO1 | 0.050793 | 0.752922 | DUSP1 | 2.790907 | 7.5E-124 |
| ULK1 | 0.029469 | 0.195163 | NOS2 | 0.012565 | 0.671149 |
| ATG3 | -0.09424 | 1.11E-06 | NCF2 | 0.010814 | 0.744004 |
| ATG4D | 0.032543 | 0.014116 | MT3 | -0.07754 | 0.136328 |
| BECN1 | -0.14309 | 0.000449 | UBC | 0.34522 | 3.32E-40 |
| MAP1LC3A | 0.034003 | 0.012779 | ALB | 0.246395 | 0.379372 |
| GABARAPL2 | -0.11334 | 0.013775 | TXNRD1 | 0.021683 | 0.831723 |
| GABARAPL1 | 0.123193 | 0.088755 | SRXN1 | 0.087691 | 0.14375 |
| ATG16L1 | -0.01359 | 0.762182 | GPX2 | -0.01383 | 0.963608 |
| WIPI1 | -0.11368 | 0.01595 | BNIP3 | -0.07232 | 0.434753 |
| WIPI2 | -0.0048 | 0.858546 | OXSR1 | 0.043325 | 0.454431 |
| SNX4 | -0.0936 | 0.219812 | ANGPTL7 | -0.00276 | 0.935253 |
| ULK2 | 0.004825 | 0.890431 | DDIT4 | 0.52818 | 1.61E-07 |
| SAT1 | 0.403431 | 8.02E-15 | ASNS | -0.07157 | 0.282486 |
| EGFR | -0.00693 | 0.680376 | TSC22D3 | 0.421685 | 8.94E-25 |
| MAPK3 | -0.07883 | 0.049218 | DDIT3 | 1.524041 | 2.78E-78 |
| MAPK1 | -0.01934 | 0.115814 | JDP2 | 0.056366 | 0.001627 |
| BID | -0.00362 | 0.873275 | SLC1A4 | -0.02282 | 0.288093 |
| ZEB1 | 0.000231 | 0.993776 | PCK2 | 0.008737 | 0.96506 |
| DPP4 | 0.033767 | 0.826522 | TXNIP | 0.195448 | 0.002903 |
| CDKN2A | 0.012467 | 0.636192 | VLDLR | -0.05494 | 0.010442 |
| PEBP1 | -0.18396 | 0.002609 | GPT2 | 0.188222 | 0.048437 |
| SOCS1 | 0.413938 | 6.4E-25 | PSAT1 | 0.123823 | 0.154974 |
| CDO1 | -0.07198 | 0.01507 | SLC7A5 | -0.11828 | 0.210639 |
| MYB | 0.011362 | 0.750871 | HERPUD1 | 0.279251 | 3E-06 |
| MAPK8 | 0.02503 | 0.25787 | XBP1 | 0.223366 | 0.000943 |
| MAPK9 | -0.05472 | 0.008215 | ZNF419 | 0.047423 | 0.031461 |
| CHAC1 | 0.009291 | 0.749598 | KLHL24 | -0.02196 | 0.840483 |
| MAPK14 | 0.013158 | 0.53617 | TRIB3 | -0.03833 | 0.420225 |
| PRKAA2 | -0.00091 | 0.979653 | ATP6V1G2 | -0.01083 | 0.693089 |
| PRKAA1 | -0.09557 | 0.012942 | VEGFA | 0.092157 | 1.59E-05 |
| ELAVL1 | -0.04137 | 0.008227 | GDF15 | 1.997537 | 1.02E-72 |
| BAP1 | -0.00538 | 0.886141 | TUBE1 | 0.016606 | 0.665203 |
| ABCC1 | -0.01141 | 0.668497 | ARRDC3 | 0.158492 | 2.38E-08 |
| ACVR1B | 0.014759 | 0.348519 | CEBPG | -0.03654 | 0.511652 |
| TGFBR1 | -0.00355 | 0.912669 | SNORA16A | 0.025228 | 0.303503 |
| EPAS1 | 0.021031 | 0.845272 | RGS4 | -0.01998 | 0.520919 |
| HIF1A | 0.108262 | 0.422676 | LOC390705 | 0.017826 | 0.480965 |
| IFNG | 0.074305 | 4.05E-05 | EIF2S1 | -0.02977 | 0.429324 |
| ANO6 | -0.17004 | 2.46E-05 | IL6 | 0.619285 | 7.5E-23 |
| LPIN1 | -0.0246 | 0.853855 | CXCL2 | 1.61395 | 1.78E-68 |
| HMGB1 | -0.06895 | 0.124025 | RELA | 0.055878 | 0.010514 |
| TNFAIP3 | 0.938647 | 2.22E-47 | HSD17B11 | -0.10137 | 0.149901 |
| TLR4 | 0.083109 | 2.87E-07 | AGPAT3 | -0.00674 | 0.894783 |
| ATF3 | 1.453296 | 1.1E-149 | SETD1B | 0.001127 | 0.971506 |
| ATM | -0.04405 | 0.003522 | FTL | 0.083543 | 0.340194 |
| YY1AP1 | 0.017878 | 0.283888 | MAFG | 0.065969 | 4.8E-07 |
| EGLN2 | -0.00295 | 0.934212 | IL33 | -0.00794 | 0.799671 |
| MIOX | -0.00305 | 0.989275 | HAMP | -0.01659 | 0.764985 |
| TAZ | -0.00352 | 0.87438 | STEAP3 | -0.02004 | 0.324839 |
| MTDH | -0.04743 | 0.594583 | DRD5 | 0.022381 | 0.440992 |
| IDH1 | -0.0468 | 0.754225 | DRD4 | 0.004007 | 0.911949 |
| SIRT1 | 0.231008 | 4.41E-08 | MAP3K5 | -0.00291 | 0.963608 |
| FBXW7 | 0.034588 | 0.002489 | SLC2A1 | 0.083927 | 0.443998 |
| PANX1 | 0.004709 | 0.885594 | SLC2A3 | 1.955936 | 2.32E-94 |
| DNAJB6 | -0.02146 | 0.795009 | SLC2A6 | -0.05827 | 0.027996 |
| BACH1 | 0.015475 | 0.461544 | SLC2A8 | -0.05459 | 0.307164 |
| LONP1 | 0.019426 | 0.795815 | SLC2A12 | -0.02988 | 0.379135 |
| SLC7A11 | 0.004744 | 0.888928 | SLC2A14 | 0.166869 | 5.6E-21 |
| GPX4 | -0.0891 | 0.222835 | EIF2AK4 | 0.083963 | 0.392424 |
| AKR1C1 | 0.024276 | 0.296103 | TFAP2C | -0.03513 | 0.396349 |
| AKR1C2 | -0.07065 | 0.001506 | SP1 | -0.02014 | 0.699483 |
| AKR1C3 | -0.17467 | 0.172115 | HBA1 | 0.943448 | 3.22E-16 |
| RB1 | -0.03493 | 0.197475 | NNMT | 0.178586 | 0.100403 |
| HSPB1 | 0.817554 | 2.61E-37 | HIC1 | 0.025512 | 0.368869 |
| HSF1 | 0.015631 | 0.557378 | STMN1 | -0.01788 | 0.261023 |
| GCLC | 0.014168 | 0.811015 | RRM2 | -0.00774 | 0.783772 |
| NFE2L2 | 0.171635 | 0.04479 | CAPG | -0.12104 | 0.002503 |
| SQSTM1 | 0.22786 | 0.000146 | HNF4A | 0.03015 | 0.188639 |
| NQO1 | -0.01391 | 0.889414 | NGB | 0.005508 | 0.868504 |
| FTH1 | 0.009243 | 0.96472 | YWHAE | -0.04397 | 0.081668 |
| MUC1 | -0.05778 | 0.641195 | GABPB1 | 0.013098 | 0.514441 |
| SLC3A2 | 0.244664 | 0.000277 | AURKA | -0.03187 | 0.04479 |
| MT1G | 0.194374 | 0.437151 | RIPK1 | 0.034098 | 0.317474 |
| SLC40A1 | -0.15536 | 0.005546 | PRDX1 | -0.22478 | 4.17E-07 |
| CISD1 | -0.18967 | 0.000386 | MIR30B | 0.013462 | 0.676085 |
| FANCD2 | -0.01453 | 0.461544 |  |  |  |

Supplementary Table S4: Identification of eight DFRGs in renal IRI.

| DFRG | logFC | adj.P.Val |
| --- | --- | --- |
| ATF3 | 1.453 | 1.131E-149 |
| JUN | 3.419 | 5.410E-163 |
| ZFP36 | 2.838 | 6.715E-122 |
| DUSP1 | 2.791 | 7.528E-124 |
| DDIT3 | 1.524 | 2.779E-78 |
| GDF15 | 1.998 | 1.016E-72 |
| CXCL2 | 1.614 | 1.781E-68 |
| SLC2A3 | 1.956 | 2.322E-94 |

| Supplementary Table S7: Association of clusters with clinical traits | | | |
| --- | --- | --- | --- |
| Clinical traits | Cluster | | P value |
|  | pBECN1(No.) | pNF2(No.) |  |
| **DGF** |  |  | 0.001 |
| No | 69 | 42 |  |
| Yes | 35 | 56 |  |
| **Donor type** |  |  | 0.004 |
| DBD | 61 | 44 |  |
| DCD | 22 | 42 |  |
| Living | 21 | 12 |  |
| The Chi-square test was performed to analyze the association of clusters with DGF and donor type. DGF, delayed graft function; DBD, donation after brain death; DCD, donation after cardiac death | | | |

| Supplementary Table S8: GSVA analysis in terms of metabolic signatures between pBECN1 cluster and pNF2 cluster. | | |
| --- | --- | --- |
| Term | logFC | adj.P.Val |
| Methionine Cycle | 0.419199 | 3.96E-37 |
| Polyamine Biosynthesis | 0.563204 | 6.77E-32 |
| Pyrimidine Metabolism | 0.399139 | 1.05E-31 |
| Purine Biosynthesis | 0.534632 | 1.89E-31 |
| Selenocompound Metabolism | 0.552582 | 3.74E-31 |
| Purine Metabolism | 0.340256 | 3.74E-31 |
| Homocysteine Biosynthesis | 0.449762 | 1.16E-29 |
| N-Glycan Biosynthesis | 0.438449 | 2.13E-25 |
| Cysteine and Methionine Metabolism | 0.3815 | 8.42E-23 |
| ADP-Ribosylation | 0.314856 | 2.23E-22 |
| Amino Sugar and Nucleotide Sugar Metabolism | 0.383319 | 3.03E-22 |
| Inositol Phosphate Metabolism | 0.298072 | 1.85E-21 |
| Glycogen Degradation | 0.373409 | 6.87E-21 |
| Terpenoid Backbone Biosynthesis | 0.461839 | 7.38E-21 |
| Starch and Suctose Metabolism | 0.307176 | 1.12E-20 |
| Glycogen Biosynthesis | 0.328952 | 1.53E-19 |
| Galactose Metabolism | 0.248124 | 8.37E-19 |
| Pentose Phosphate | 0.31788 | 1.41E-16 |
| Fructose and Mannose Metabolism | 0.291373 | 2.51E-16 |
| Ubiquinone and other Terpenoid-Quinone Biosynthesis | 0.472672 | 6.59E-16 |
| Glycosphosphatidylinositol | 0.395421 | 6.59E-16 |
| Arginine and Proline Metabolism | 0.308419 | 5.57E-15 |
| Glutathione Metabolism | 0.301866 | 1.57E-14 |
| Nicotinamide Adenine Metabolism | 0.358362 | 7.96E-14 |
| Linoleic Acid Metabolism | -0.23252 | 8.72E-14 |
| Shingolipid Metabolism | 0.224005 | 8.61E-12 |
| Citric Acid Cycle | 0.408468 | 1.9E-11 |
| Oxidative Phosphorylation | 0.390174 | 5.35E-11 |
| Glycosaminoglycan Degradation | 0.285281 | 9.84E-11 |
| Glycolysis | 0.22204 | 1.23E-10 |
| Pyruvate Metabolism | 0.315146 | 1.72E-10 |
| Gluconeogenesis | 0.243234 | 2.48E-10 |
| Other Glycan Degradation | 0.311141 | 3.43E-10 |
| Pyrimidine Biosynthesis | 0.276457 | 4.79E-10 |
| Folate One Carbon Metabolism | 0.300411 | 1.19E-09 |
| Urea Cycle | 0.269416 | 2.36E-09 |
| Other Types of O-Glycan Biosynthesis | 0.209941 | 2.39E-09 |
| Cholesterol Biosynthesis | 0.35292 | 2.53E-09 |
| Fatty Acid Elongation | 0.297201 | 5.37E-09 |
| Propanoate Metabolism | 0.35252 | 1.61E-08 |
| Pantothenate and CoA Biosynthesis | 0.247578 | 9.14E-07 |
| Steroid Biosynthesis | 0.249884 | 1.99E-06 |
| Biosynthesis of Unsaturated Fatty Acids | 0.26086 | 2.25E-06 |
| Lysine Degradation | 0.202643 | 2.4E-06 |
| Valine, Leucine and Isoleucine Degradation | 0.298653 | 5.89E-06 |
| Fatty Acid Degradation | 0.240013 | 2.98E-05 |
| Beta-Alanine Metabolism | 0.227176 | 0.000116 |
| Glyoxylate and Dicarboxylate Metabolism | 0.253842 | 0.00038 |

Supplementary Figure 1: Protein and protein interaction of top 10 correlated genes in BECN1 cluster.


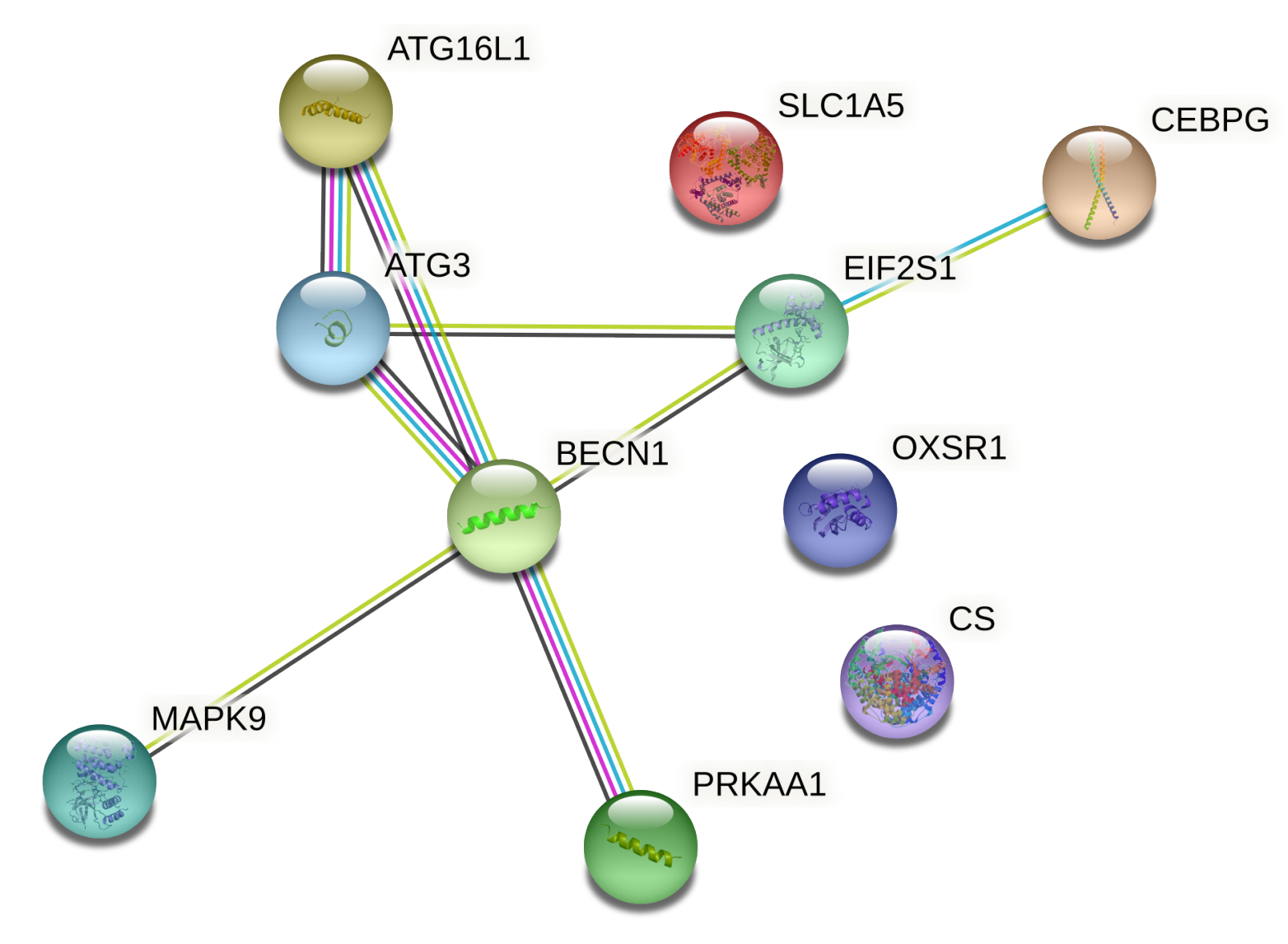


Supplementary Figure 2: GSVA analysis in terms of KEGG pathways between pBECN1 cluster and pNF2 cluster


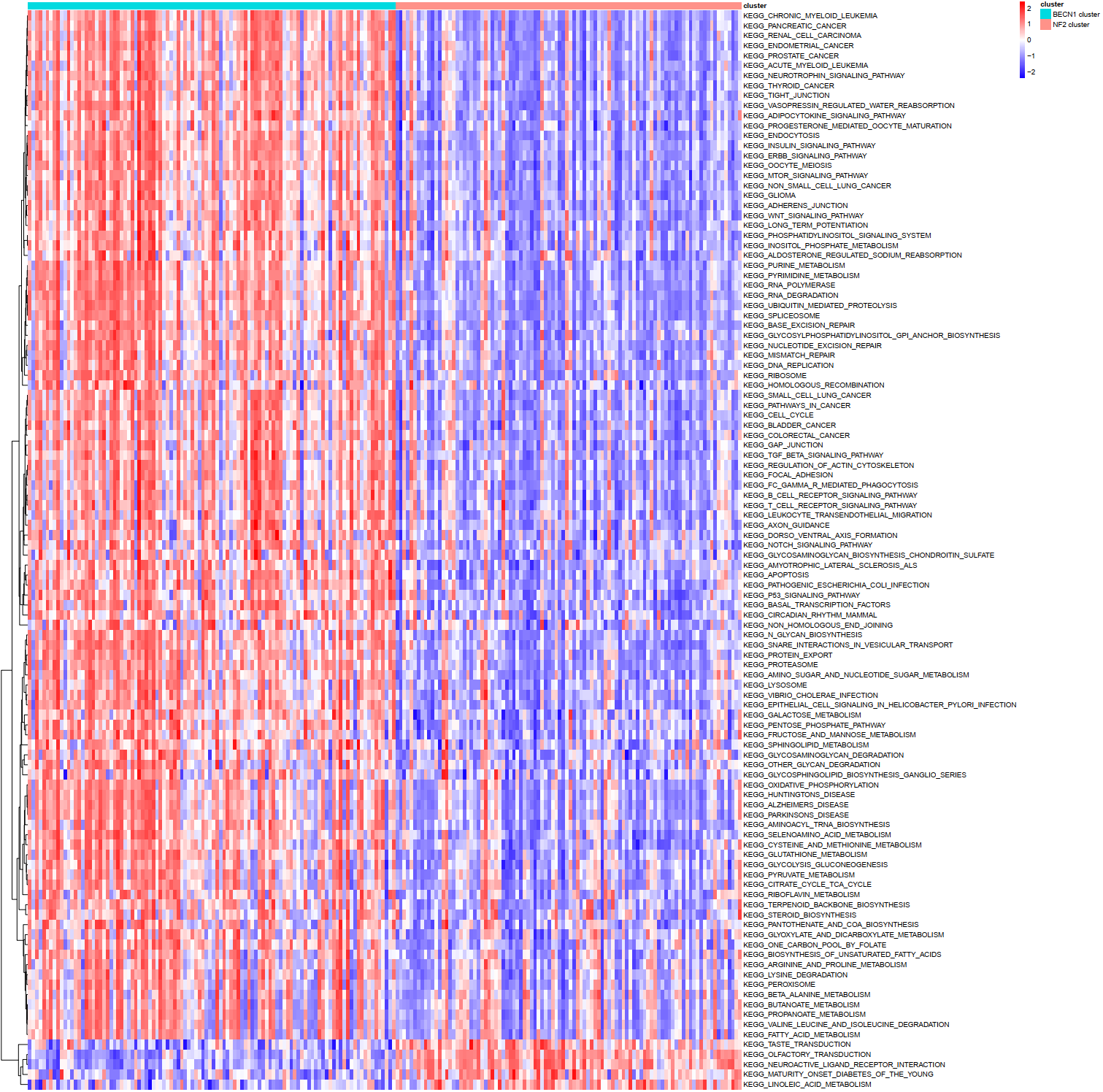

Supplement: Supplementary file 3 [file DataSheet1.docx]
